# **CRISPR/Cas9 genome editing technology in filamentous fungi: progress and perspective**

**DOI:** 10.1007/s00253-019-10007-w

**Published:** 2019-07-22

**Authors:** Runjie Song, Qing Zhai, Lu Sun, Enxia Huang, Yu Zhang, Yanli Zhu, Qingyun Guo, Yanan Tian, Baoyu Zhao, Hao Lu

**Affiliations:** 1 College of Veterinary Medicine, Northwest A&F University, Yangling, 712100 Shaanxi China; 2 Tibet Academy of Agricultural and Animal Husbandry Sciences, Lhasa, 850000 Tibet China; 3 Qinghai Academy of Agriculture and Forestry Sciences, Qinghai University/Key Laboratory of Agricultural Integrated Pest Management, Qinghai Province/State Key Laboratory of Plateau Ecology and Agriculture, Qinghai University, Xining, 810016 Qinghai China; 4 Department of Veterinary Physiology and Pharmacology, College of Veterinary Medicine, Texas A&M University, College Station, TX 77843 USA

**Keywords:** Filamentous fungi, CRISPR/Cas9, Genome editing, Off-target

## Abstract

Filamentous fungi play an important role in human health and industrial/agricultural production. With the increasing number of full genomes available for fungal species, the study of filamentous fungi has brought about a wider range of genetic manipulation opportunities. However, the utilization of traditional methods to study fungi is time consuming and laborious. Recent rapid progress and wide application of a versatile genome editing technology, i.e., the CRISPR (clustered regularly interspaced short palindromic repeat)–Cas9 (CRISPR-related nuclease 9) system, has revolutionized biological research and has many innovative applications in a wide range of fields showing great promise in research and application of filamentous fungi. In this review, we introduce the CRISPR/Cas9 genome editing technology focusing on its application in research of filamentous fungi and we discuss the general considerations of genome editing using CRISPR/Cas9 system illustrating vector construction, multiple editing strategies, technical consideration of different sizes of homology arms on genome editing efficiency, off-target effects, and different transformation methodologies. In addition, we discuss the challenges encountered using CRISPR/Cas9 technology and give the perspectives of future applications of CRISPR/Cas9 technology for basic research and practical application of filamentous fungi.

## Introduction

Filamentous fungi have widely been used for expression of heterologous proteins, and production of antibiotics and organic acid (Jiang et al. 2013; Osiewacz 2002). At present, the filamentous fungi used for production of enzymes and recombinases are mainly *Aspergillus niger*, *Aspergillus oryzae*, *Rhopzus oryzae*, and *Aspergillus nidulans*. Filamentous fungi produce abundant secondary metabolites, such as paclitaxel and swainsonine (SW), which have become important clinical therapeutics.

Filamentous fungi such as *A. nidulans* and *Neurospora crassa* are often used as model eukaryotic microorganisms and play a critical role in basic research. *N. crassa* is one of the earliest filamentous fungi used for genetic studies. *Metarhizium anisopliae*, *Pyricularia oryzae*, and *Aspergillus fumigatus* are important pathogens of animals, plants, and humans, respectively. As a multicellular eukaryotic microorganism, filamentous fungi have a genetic background that is more complex than those of bacteria and other prokaryotes, and their genetic manipulation is often difficult. This has led to a relatively slow progress in molecular biology and genetic research of filamentous fungi.

**Fig. 1:**
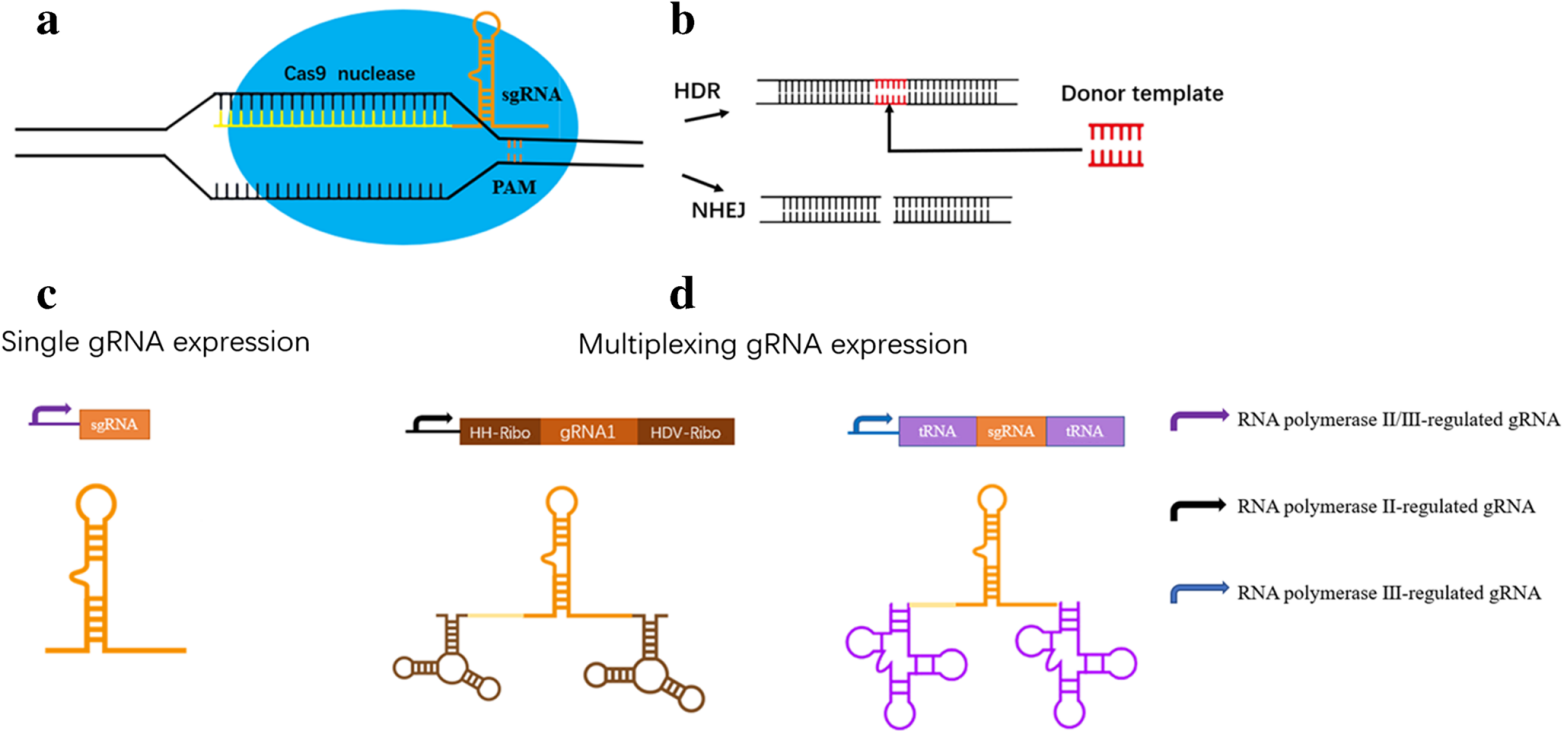
Schematic illustration of Cas9/gRNA genome editing. **a** sgRNA-mediated Cas9 protein can bind to target sequences of site and cut DNA double strands. **b** When the DNA double-strand breaks occur, the cell initiates the self-repair mechanism. The NHEJ-dominated repair pathway will cause the random loss, insertion, and replacement of bases at the breakage point, resulting in gene mutation. The HR pathway will accurately edit the target gene guided by the donor DNA fragment. **c** Single-promoter-driven gRNA expression cassette. **d** multiple gRNA expression cassettes can be constructed by concatenating 2 or more of gRNA linked together by linkers, which can then be enzymatically processed into multiple single gRNAs thus targeting multiple sites

Interestingly, the breakthrough of the manipulation of eukaryotic organisms began with exploration of the much simpler bacterial genomes through rudimentary bioinformatic analysis. Mojica et al. (2005) found, based on the available DNA sequence data base, that clustered regularly interspaced short palindromic repeats (CRISPR) loci from bacterial DNA data base matched the sequences of bacterial phage and made an important conjecture that CRISPR must be part of the bacterial immune system used by the host bacteria to guard against repeated infection of the same phage (Lander 2016). With the continuous deepening of research, accumulated information on CRISPR/Cas9 system suggest there are two categories of CRISPR/Cas9 systems, which can be further divided into 6 types and 19 subtypes (Makarova et al. 2015; Shmakov et al. 2015; Shmakov et al. 2017). Current major research results and efforts gravitated toward type II CRISPR/Cas9 system which includes Cas9 (nuclease), mature crRNA (CRISPR associate RNA), tracrRNA (trans-activating crRNA), and RNaseIII. The type II system is simpler than other CRISPR systems and has been widely used. Jinek et al. (2012) further modified and optimized the bacterial type II CRISPR system by linking crRNA and tracrRNA into a single-guide RNA (sgRNA), which can efficiently direct the Cas9 protein to the target sequences to cut the DNA. After constructed exogenous CRISPR/Cas9 system is introduced into the recipient cells, the crRNA-tracrRNA (sgRNA) complex further interacts with Cas9 to form a crRNA-tracrRNA-Cas9 complex. The Cas9 protein contains two key domains, HNH and RuvC, which cleave the two strands of the DNA through binding to the target sequences and then cleaves the DNA duplex under the guidance of sgRNA. The HNH domain can cleave the DNA strand complementary to the crRNA, and the RuvC domain is responsible for the cleavage of the DNA that is not complementary to the crRNA (Jinek et al. 2014; Nishimasu et al. 2014; Sternberg et al. 2014). Therefore, sgRNA is responsible for identifying the 20 nucleotide sequences upstream of the protospacer adjacent motif (PAM) site, and the Cas9 protein is responsible for shearing 3–4 bp upstream of PAM to form blunt ends (Fig. 1a).

When DNA double-strand breaks (DSBs) occur, the genomic DNA initiate self-repair mechanism resulting in genome mutation, non-homologous end-joining (NHEJ) as the dominant repair pathway which will cause random loss, insertion, and replacement of bases at the DNA breakage points, the homologous repairing (HR) pathway will precisely edit the gene of interest with the help of exogenous donor fragments (Sander and Joung 2014) (Fig. 1b). Currently, CRISPR/Cas9 technology has optimized and utilized for genome editing in numerous organisms (Cong et al. 2013; Mali et al. 2013a; DiCarlo et al. 2013), generating great impacts in humanity from industrial biotechnology (van Erp et al. 2015) to plant breeding (Bortesi and Fischer 2015; Schiml and Puchta 2016) and show great promise in treatment of human disease (Cai et al. 2016).

**Fig. 2:**
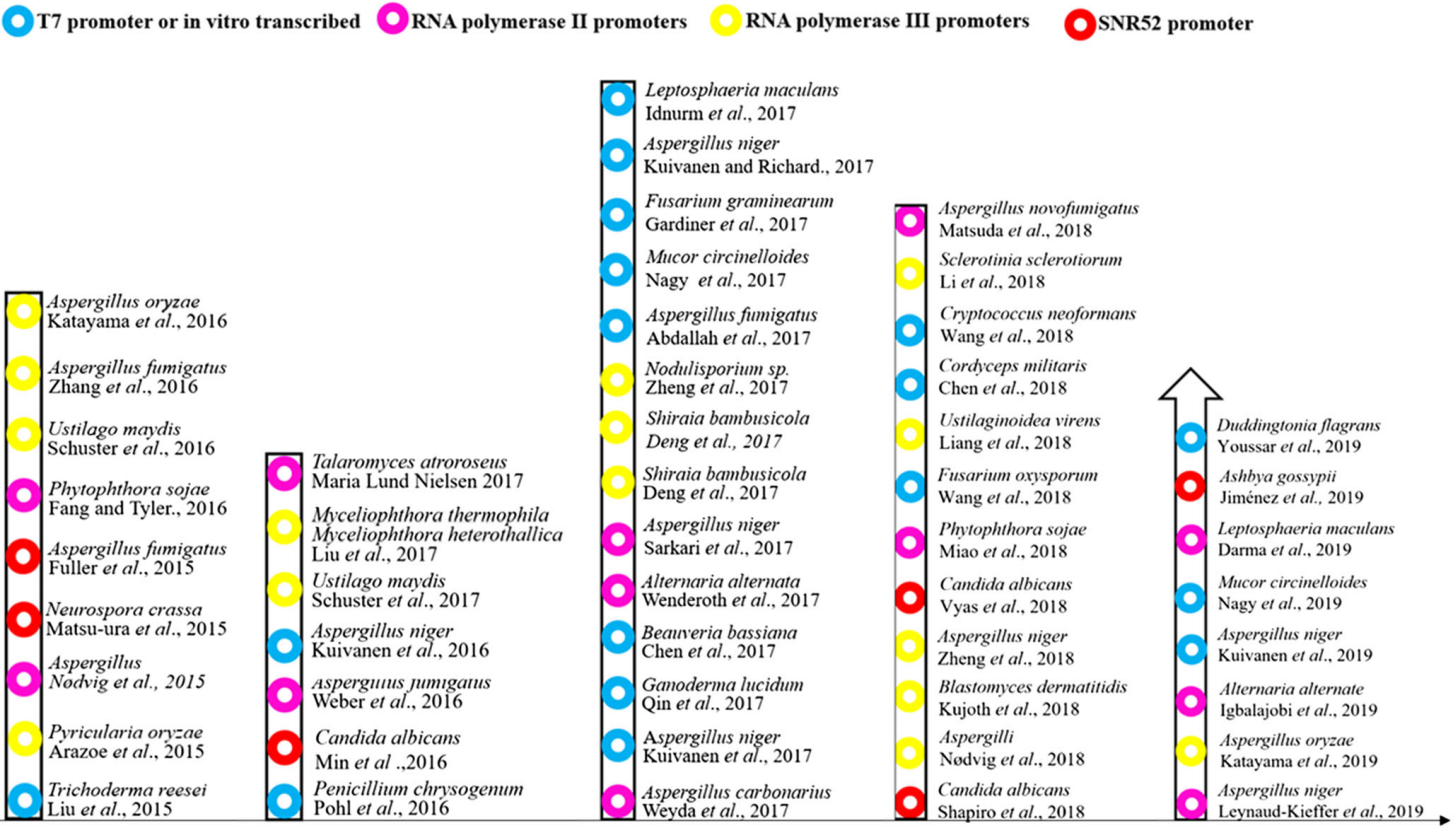
The history of the development and application of CRISPR/Cas9 technology in filamentous fungi; different colors represent different promoter-driven gRNA expression cassettes

This review covers the latest developments in application of CRISPR/Cas9-mediated genome editing in filamentous fungi (Fig. 2), highlighting the general technical issues of using CRISPR/Cas9-based genome editing approaches in fungi (Grzybek et al. 2018; Wang et al. 2017; Shi et al. 2017). In addition, future development and challenges of CRISPR/Cas9 technology are discussed.

## CRISPR/Cas9 genome editing in filamentous fungi

In recent years, the birth of CRISPR/Cas9 genome editing technology has shown great promise to revolutionize the field of fungal research. Dicarlo et al. (2013) first introduced the CRISPR/Cas9 genome editing system into *Saccharomyces cerevisiae*. Subsequently, Liu et al. (2015) applied the CRISPR/Cas9 genome editing system to *Trichoderma reesei*; Matsu-ura et al. (2015) and Nodvig et al. (2015) applied this system to the model fungus *N. crassa* and *A. nidulans*, respectively. Since then, the CRISPR/Cas9 genome editing system has found wide applications genetically alteration of many filamentous fungi (Table 1)*.*

**Table 1:** The application of the CRISPR/Cas9 system in filamentous fungi

Species	Cas9 expression (selection marker, promoter)	Delivery method	Editing method	Application and efficiency	Reference
*T. reesei*	Codon-optimized Cas9, *ura5*, *pdc/cbh1*	PMT	NHEJ/HDR	Single/multiple-gene disruption ⩾ 93%/4.2–45%	Liu et al. 2015
*P. oryzae*	Codon-optimized Cas9, *Bar*, *tef1*	PMT	NHEJ/HDR	Single-gene disruption, 36.1–80.5%	Arazoe et al. 2015
*Aspergillus*	pFC332, *pyrG/argB/hph/ble*, *gpdA*	PMT	NHEJ	Single-gene disruption	Nodvig et al. 2015
*N. crassa*	Codon-optimized Cas9, *Bar*, *Trpc*	PMT	NHEJ/HDR	Single-gene disruption	Matsu-Ura et al. 2015
*A. fumigatus*	Human codon-optimized Cas9, *hph/ble*, *amy*	PMT	NHEJ	Single-gene disruption, 25%-53%	Fuller et al. 2015
*P. sojae*	Human-optimized codons Cas9, *G418*, *Ham34*	PMT	NHEJ/HDR	Single-gene disruption	Fang and Tyler 2016
*U. maydis*	Codon-optimized Cas9, *ip*, *Otef* (modified tef1)/*hsp70*,	PMT	NHEJ	Single/double-gene disruption, 50%-90%	Schuster et al. 2016
*A. fumigatus*	Human codon-optimized Cas9, *pyr4/hph*, *niiA/gpdA*	PMT	HDR	Single/double-gene disruption, 95%-100%	Zhang et al. 2016
*A. oryzae*	Codon-optimized Cas9, *pyrG*, *amyB*	PMT	NHEJ	Single-gene disruption, 10–20%	Katayama et al. 2016
*P. chrysogenum*	Human codon-optimized Cas9, *amdS*, *xlnA*	PMT	NHEJ/HDR	Single-gene disruption, 100%	Pohl et al. 2016
*C. albicans*	Codon-optimized Cas9, *Nat*, *ENO1*	Lithium acetate	NHEJ/HDR	Single-gene disruption	Min et al. 2016
*A. fumigatus*	pFC332, *pyrG*, *TetON*	PMT	NHEJ/HDR		Weber et al. 2017
*A. niger*	pFC332, *pyrG/hph*, *tef1*	PMT	NHEJ/HDR	Single-gene disruption, 37.5–100%	Kuivanen et al. 2016
*U. maydis*	Codon-optimized Cas9, *ip*, *Otef* (modified tef1)/*hsp70*	PMT	NHEJ	Multiple-gene disruption, 50–90%	Schuster et al. 2018
*M. thermophila* *M. heterothallica*	Codon-optimized Cas9, *Bar*, *tef1*	PMT/AMT	NHEJ/HDR	Single/multiple-gene disruption, 15–95%	Liu et al. 2017
*T. atroroseus*	pFC330, *hph*, *tef1*	PMT	NHEI	Single-gene disruption	Nielsen et al. 2017
*A. carbonarius*	pFC332, *hph*, *tef1*,	AMT	NHEJ/HDR	Single-gene disruption, 27%	Weyda et al. 2017
*A. niger*	pFC332, *hph*, *tef1*	PMT	NHEJ	Single-gene disruption	Kuivanen et al. 2017
*Ganoderma lucidum*	Codon-optimized Cas9, *ura3*, *gpdA*	PMT	NHEI	Single-gene disruption,	Qin et al. 2017
*B. bassiana*	Codon-optimized Cas9, *gfp/ura5/bar*, *gpdA*	PMT	NHEJ/HDR	Single/multiple-gene disruption, 5–50%	Chen et al. 2017
*A. alternata*	pFC332, *pyr4/hph*, *gpdA*	PMT	NHEJ	Single-gene disruption	Wenderoth et al. 2017
*S. bambusicola*	Codon-optimizedCas9, *hph*, *TrpC*	PMT	NHEJ	Single-gene disruption	Deng et al. 2017a
*S. bambusicola*	Codon-optimizedCas9, *hph*, *TrpC*	PMT	NHEJ/HDR	Single-gene disruption, 32%	Deng et al. 2017b
*Nodulisporium* sp.	Codon-optimized Cas9, *Bar*, *TrpC*	PMT	NHEJ/HDR	Single-gene disruption	Zheng et al. 2017
*Mucor circinelloides*	SpCas9, *pyr4*	PMT	NHEJ/HDR	Single/double-gene disruption, 100%	Nagy et al. 2017
*Fusarium graminearum*	Codon-optimized Cas9, fludioxonil, *gpdA*	PMT	NHEJ/HDR	Single-gene disruption, 1–10%	Gardiner and Kazan 2018
*Leptosphaeria maculans*	Human codon-optimized Cas9, *Ip/G418/hph*, *act1*	AMT	NHEJ	Single-gene disruption	Idnurm et al. 2017
*A. niger*	pFC332, *hph*, *tef1*	PMT	NHEJ	Single-gene disruption	Kuivanen and Richard 2018
*C. albicans*	SpCas9, *Nat/Phloxine B*	Electroporation	NHEJ	Double-gene disruption, 53–98%	Shapiro et al. 2018
*Aspergilli*	pFC332, argB/pyrG, tef1	AMT	HDR	Multiple-gene disruption, 15–90%	Nodvig et al. 2018
*B. dermatitidis*	pFC332, *hph*, *tef1*	AMT	NHEJ	Single/double-gene disruption, 22–73%	Kujoth et al. 2018
*A. niger*	Codon-optimized Cas9, *hph/amdS*, *glaA*	PMT	NHEL/HDR	Single-gene disruption, 33.3–100%	Zheng et al. 2018
*C. albicans*	Codon-optimized Cas9, *Nat*, *ENO1*	Electroporation	HDR	Single/double-gene disruption, 25–100%	Vyas et al. 2018
*P. sojae*	Human-optimized codons Cas9, *G418*, *Ham34*	PMT	NHEJ	Double-gene disruption	Miao et al. 2018
*F. oxysporum*	pFC332, *hph*	PMT	NHEJ/HDR	Single-gene disruption, 20–53.8%	Wang et al. 2018
*Ustilaginoidea virens*	Codon-optimized Cas9, *G418*, *pdc/cbh1*	AMT PMT	NHEJ	Multiple-gene disruption, 60%-90%	Liang et al. 2018
*Cordyceps militaris*	Codon-optimized Cas9, *5-FOA/blpR*, *tef1*	AMT/PMT	NHEJ/HDR	Single-gene disruption, 87.2–84.3%	Chen et al. 2018
*Cryptococcus neoformans*	Codon-optimized Cas9, *Ntc*, *tef1*	Electroporation	HDR	Single-gene disruption, 96.5–100%	Wang 2018
*Sclerotinia sclerotiorum*	Codon-optimized Cas9, *hph*, *tef1*	PMT	NHEJ/HDR	Single-gene disruption, 38–100%	Li et al. 2018
*Aspergillus novofumigatus*	pFC332, *pyrG*, *tef1*	PMT	NHEJ	Single-gene disruption	Matsuda et al. 2018
*A. niger*	pFC332, argB/pyrG, *tef1*	PMT	HDR	Single-gene disruption, 100%	Leynaud-Kieffer et al. 2019
*A. oryzae*	Codon-optimized Cas9, *pyrG*, *amyB*	PMT	HDR	Single/double-gene disruption, 50–100%	Katayama et al. 2019
*A. alternata*	pFC332, *hph*, *tef1*	PMT	NHEJ	Single-gene disruption	Igbalajobi et al. 2019
*A. niger*	Codon-optimized Cas9, *pyrG*	PMT	NHEJ/HDR	Single/multiple-gene disruption 100%	Kuivanen et al. 2019
*M.r circinelloides*	SpCas9, *pyr4*	PMT	HDR	Multiple-gene disruption 100%	Nagy et al. 2019
*L. maculans*	Human codon-optimized Cas9, *hph*, *act1*	AMT	NHEJ	Double-gene disruption	Darma et al. 2019
*A. gossypii*	Human-optimized codons Cas9, *G418*, *tef1*	Electroporation	NHEJ	Single-gene disruption, 44–85%	Jiménez et al. 2019
*Duddingtonia flagrans*	pFC332, *hph*, *tef1*	PMT	HDR	Single-gene disruption	Loubna et al. 2019

## Cas9 expression

The Cas9 protein is an important component of the CRISPR/Cas9 system that performs endonuclease function and has a total length of approximately 1400 amino acids. The CRISPR/Cas9 system was discovered in bacteria or Archaea. Therefore, when the CRISPR/Cas9 system is used in fungi, genomes encoding the Cas9 protein are usually fungal codon-optimized and a nuclear localization signal is added at both ends of the Cas9 gene. Therefore, amino acid sequence of Cas9 can be bacterial codon-optimized (Liu et al. 2015), human codon-optimized (DiCarlo et al. 2013; Fuller et al. 2015; Fang and Tyler 2016; Zhang et al. 2016; Pohl et al. 2016; Idnurm et al. 2017), or fungal codon-optimized (Nodvig et al. 2015; Nodvig et al. 2018; Generoso et al. 2016). The classical nuclear localization sequence (NLS) SV40 has been shown to function in many organisms (Nodvig et al. 2015). However, Wang et al. (2018) and Fang and Tyler (2016) demonstrated that this nuclear localization signal is not applicable to *Fusarium oxysporum* and *Phytophthora sojae*, for which they used an endogenous nuclear localization signal to fuse with Cas9 and successfully applied to *F. oxysporum* and *P. sojae*. In order to detect the expression and localization of optimized Cas9, most of the studies have fused the enhanced green fluorescent protein (eGFP) gene to the optimized Cas9. Thereafter, it was verified whether the Cas9 protein was expressed in the recipient fungus by detecting fluorescence (Fang and Tyler 2016; Wang et al. 2018; Chen et al. 2017; Chen et al. 2018).

The successful expression of the Cas9 gene depends on the types of promoter used. The strength of the promoter in driving transcription is an important factor affecting the expression efficiency of Cas9 exogenous genes, so the selection of a suitable promoter is of great significance for the function of the CRISPR/Cas9 system. Constitutive promoters are the most common and are not induced by external factors. It is a continuous expression system in all tissues and organs in an organism. The RNA transcription and protein expression levels of such promoter-initiated genes are relatively stable and have no spatial or temporal specificity. In general, most studies have used constitutive promoters to drive expression of Cas9 protein in filamentous fungi, such as the *trpC* promoter of the *A. nidulans* tryptophan synthesis gene *trpC*, the *gpdA* promoter of the *A. nidulans* glyceraldehyde-3-phosphate dehydrogenase gene *gpdA*, and *TEF1* the promoter of the *A. nidulans* translation elongation factor 1α (Matsu-Ura et al. 2015; Nodvig et al. 2015; Arazoe et al. 2015; Sarkari et al. 2017). In addition to the three common promoters (*trpC*, *gpdA* and *TEF1*), *xlnA*, *Ham34*, *amyB*, *niiA*, *Otef* (modified tef1), and *hsp70* have also been successfully used for Cas9 expression in filamentous fungi (Zhang et al. 2016; Pohl et al. 2016; Katayama et al. 2016; Schuster et al. 2018). If mutants generated using the CRISPR system are used in studies of pathogenesis and secondary metabolites, expression of the Cas9 nuclease itself cannot exert adverse effects on the recipient strain. However, studies have shown that the expression of Cas9 as a foreign gene in recipient organisms may have negative effects (Miyagishi and Taira 2002). Therefore, it is important to ensure that Cas9 protein has no adverse effect on filamentous fungi by testing the Cas9 expression strain’s growth, development, stress resistance, and virulence and other biological characteristics (Schuster et al. 2016; Deng et al. 2017a; Deng et al. 2017b).

## Guide RNA expression

### RNA polymerase III promoter–regulated gRNA

The gRNA in the CRISPR/Cas9 system does not contain a cap structure and a poly A tail, and requires a clear transcription starting point. In most cases, gRNA is driven by RNA polymerase type III *U6* promoters that have been shown to have base preference and persistence in driving transcription (Gao and Zhao 2014). Bioinformatic analysis has shown that *U6 snRNA* gene sequences are highly conserved from yeast to mammals. Arazoe et al. (2015) first compared human *U6 snRNA* and analyzed two endogenous *U6* promoters of *P. oryzae* and successfully knocked out *P. oryzae*’s *SRS2* gene with an efficiency of 36.1–80.5%. CRISPR/Cas9 system with *U6* promoter to transcribe gRNA has been used for genetic manipulation in most filamentous fungi (Fig. 1c). The gRNA in the CRISPR/Cas9 system requires a well-defined transcription start site, and the transcription of gRNA in higher organisms is usually driven by the Pol III *U6* promoter. In addition to the *U6* promoter, Schuster et al. (2018) optimized the CRISPR/Cas9 system and tested the expression efficiency of *tRNA* promoter–driven gRNA (ptRNALeu-TAA, ptRNAGlyGCC, ptRNATyrGTA, and ptRNAGlyTCC). The four *tRNA* promoters have higher driving efficiencies than the *U6* promoter, indicating that *tRNA* promoters can be used for gRNA expression as well (Liang et al. 2018).

However, the genome editing efficiency of different *tRNA* promoters varies greatly in a strain-dependent manner. In some cases, *tRNA*-based genome editing efficiency remains controversial. In *Yarrowia lipolytica*, the use of *tRNA* as the sgRNA promoter brings about 30% gene fragmentation efficiency (Schwartz et al. 2016). Therefore, *tRNA* is preferably used to excise sgRNA from the primary transcript by its endogenous processing mechanism, rather than acting as a promoter. In order to find a more efficient promoter, Zheng et al. (2018) found that *5S rRNA* is a basic cellular component that is highly conserved and abundant in cells. Therefore, they used the *5S rRNA* gene of *A. niger* and its 338-bp upstream sequence as a promoter and fused with the sgRNA sequence to construct an sgRNA expression cassette. Meanwhile, in order to avoid the interference of *5S rRNA* on the structure and function of sgRNA, an 88-bp HDV ribozyme gene from *Trichoderma atroviride* was added between the *5S rRNA* gene and sgRNA. Experiments have shown that the CRISPR/Cas9 system based on the *5S rRNA* promoter shows a gene disruption efficiency close to 96%, which is significantly higher than the 15% and 23% destruction efficiency based on the *PhU6* or *PanU6* promoter system. The selection of promoters for sgRNA expression represents an important technical limitation in the development of the CRISPR/Cas9 genome editing system.

### RNA polymerase II–regulated gRNA

The gRNAs such as those produced from expression from the *U3* and *U6* promoter are transcribed using RNA polymerase III (pol III) in most organisms. However, the *U6* or *U3* promoter transcribes gRNA with many limitations. Firstly, *U6 snRNA* and *U3 snRNA* are housekeeping genes that are ubiquitously expressed. Therefore, they cannot be used to produce cell type or tissue-specific gRNA. Secondly, *U6* and *U3* promoters are not suitable for conventional in vitro transcription of gRNA because RNA polymerase III is not commercially available. In addition, the CRISPR target sequences recognized by the *U6* and *U3* promoter are constrained with certain sequence specificity and must be G(N)20GG and A(N)20GG (Gao and Zhao 2014; Nissim et al. 2014).

Gao and Zhao (2014) used the nuclease activity of ribozymes to design the artificial gene RGR (ribozyme-gRNA-ribozyme), a 5′-terminal hammerhead (HH) and a 3′-terminal hepatitis D virus (HDV), which flank sgRNA. This system was introduced into yeast under the control of the RNA polymerase II–transcribed alcohol dehydrogenase 1 (*ADH1*) promoter to achieve targeted DNA cleavage. It has been shown that in this system targeting sequence is no longer limited to G(N)20GG or A(N)20GG. Subsequently, Nodvig et al. (2018) first constructed ribozyme-gRNA-ribozyme using the *A. nidulans* strong constitutive *gpdA* promoter (*PgpdA*) and the *trpC* terminator (*TtrpC*), and successfully applied it to species in the filamentous fungus genus *Aspergillus*. Wenderoth et al. (2017) knocked out the key genes *pksA* and *brm2* controlling the melanin biosynthetic pathway in *Alternaria alternata* using the same method and successfully obtained gene mutant strains. In order to optimize Cas9 and gRNA expression, Nodvig et al. (2018) introduced the strong constitutive promoter *gpdA* and *tef1*-controlled *mRFP* into the NHEJ deletion strain *NID1*; in comparison, it was found that the *tef1* promoter is more efficient than *gpdA* promoter. Recently, Kujoth et al. (2018) adapted RNA Pol II system to achieve double gene knockout on *Blastomyces dermatitidis* by *Agrobacterium*-mediated transformation. It is further proven that the knock-out efficiency of the single-promoter-driven double gRNA is higher than that of dual-promoter.

However, the aforementioned methods are relatively complex and difficult to operate and may increase the burden on the carrier and may even bring additional foreign substances in the cells to cause toxic effects. Subsequently, Wang et al. (2015) used the Golden Gate method (another RNA Pol II–driven system) to design a miRsh-sgRNA cassette that is regulated by an RNA polymerase II promoter. The miRsh-sgRNA cassette has been experimentally proven to be effective in expressing gRNA. The gRNA cassette could generate gRNA targeting the *p53* gene and achieve specific cleavage of the *P53* target gene under Cas9 protein mediated. This miRsh-sgRNA structure regulated by the RNA polymerase class II promoter allows for more controlled and safer gene editing in the future using the CRISPR/Cas9 system. This effective method has not been used in the filamentous fungi, and its utility in the filamentous fungi warrants further investigation.

### Other strategies for generating gRNA

In addition to the above methods, two methods can be used to generate functional gRNA. One strategy is by using the *SNR52* promoter of *S. cerevisiae*, which was shown to transcribe the initial gRNA with a leader sequence that can be excised during processing to form a functional gRNA (Matsu-Ura et al. 2015; Fuller et al. 2015). In addition, the plasmid-free CRISPR-Cas9 system is also capable of genome editing of fungal cells.

The plasmid-free CRISPR-Cas9 system assembles Cas9 protein and sgRNA to form a stable RNP in vitro, and transfers the complex to fungal protoplasts by PEG- or electroporation-mediated methods for genome editing. There are several advantages to using this system for genome editing compared with plasmid transformation. Cas9 and sgRNA are capable of forming stable ribonucleoproteins in vitro, and thus, RNA degradation is less likely than plasmid transformation. In addition, the method is capable of assessing the target cleavage efficiency of different sgRNAs in vitro, so efficient sgRNA can be selected for research. This RNP-mediated genome editing technique has been successfully applied to some species (Min et al. 2016; Grahl et al. 2017; Wang 2018; Al Abdallah et al. 2017; Kuivanen and Richard 2018).

### Multiplexing gRNA expression

The above reports all used CRISPR/Cas9 technology to knock out a single gene, but in practical applications, some fungal traits are usually controlled by multiple genes. There is a great need for simultaneous knockouts of multiple genes, and the traditional methods for obtaining multiple gene mutants are time consuming and laborious. The cumbersome ZFN and TALEN technologies are also limited in the ability to knock out multiple genes at the same time. In contrast, the CRISPR/Cas9 technology can be used to knock out multiple genes.

The core problem of knockout of multiple genes lies in multiple targeting sites. Therefore, this can be eliminated as long as the problem of multiple gRNA expression cassettes is solved. Currently, multi-gene simultaneous targeting mainly focuses on the following three methods: (1) Liu et al. (2015) introduced the vector containing the Cas9 expression cassette into the recipient fungal cells by the *Agrobacterium*-mediated method, and then introduced multiple mature gRNA synthesized in vitro into the Cas9-positive cells through protoplast-mediated methods. This method has been applied to *A. fumigatus* (Zhang et al. 2016), *A. niger* (Kuivanen et al. 2016), and *Beauveria bassiana* (Chen et al. 2017). (2) The *tRNA*-spacer system has been successfully used for multiple sgRNA expression in other organisms, including plants, yeast, human cells, and fruit flies (Xie et al. 2015; Schwartz et al. 2016; Port and Bullock 2016; Qi et al. 2016; Dong et al. 2017). Specifically, the gRNAs are concatenated, interspaced by 5′ and 3′ splice sites of a tRNA that are recognized by RNase P and RNase Z. The mature gRNAs are then released through the action of RNase P and RNase Z (Fig. 1d). Nodvig et al. (2015) used this mechanism to construct a multiple gRNAs expression vector and verified that the polymerase III promoter *PAf-U3* was more efficient than *PgpdA*. (3) In addition, Kujoth et al. (2018) knocked out two genes, *PRA1* and *ZRT1*, of *B. dermatitidis* using two gRNA expression cassettes driven by the RNA polymerase II promoter *PgpdA* (Fig. 1d).

## Delivery of the Cas9 and sgRNA expression cassettes into fungal cells

CRISPR/Cas9 system targeting fungal genome must be delivered to fungal cells by vectors carrying Cas9 and sgRNA expression cassettes. Typically, the Cas9 protein expression cassette and the sgRNA expression cassette are carried by a single vector or a double vector. Zhang et al. (2016) validated the efficiency of two different methods of expression in the model fungus *A. fumigatus.* They show that the accuracy and efficiency of using a single-vector expression system is significantly higher than that of a dual-vector expression system. This may be due to the fact that the ratio of Cas9 and sgRNA expression cassettes transformed into fungal cells cannot be precisely controlled. Therefore, most studies first stably transduced Cas9 expression cassettes into recipient cells and screened for positive strains expressing Cas9 protein; then, the Cas9-positive cells are transfected with the in vitro transcribed gRNA for genome editing (Liu et al. 2015; Chen et al. 2017; Kuivanen et al. 2016; Kuivanen et al. 2017). The polyethylene glycol (PEG) transformation and *Agrobacterium*-mediated transformation (AMT) are the two most common methods for transforming CRISPR/Cas9 systems into fungal cells. Since PEG-mediated transformation methods are the easiest for many fungi, most studies use this method to transform Cas9 and gRNA expression cassettes into fungal cells (Li et al. 2018; Matsuda et al. 2018; Miao et al. 2018). *Agrobacterium*-mediated traditional genome editing is also a common method. However, this method is not common in CRISPR/Cas9 system–mediated fungal genome editing. Recently, Weyda et al. (2017) compared these two approaches and demonstrated that *Agrobacterium*-mediated CRISPR/Cas9 systems can also be used for efficient genome editing in fungal cells.

## Regulation of NHEJ or HR pathways

DSBs generated by Cas9 can be directly subjected to indel mutagenesis by non-homologous end-joining repair (NHEJ); or homology repair (HR) can be achieved if a DNA repair template (donor DNA) is provided (Ma et al. 2016; Doudna and Charpentier 2014; Shalem et al. 2015). The NHEJ repair route is different from the HR repair pathway (Fig. 1b). The poor fidelity of the NHEJ repair pathway can occur in the G1 phase of the entire cell cycle, and HR repair is a precise repair pathway that occurs only during DNA replication, and the NHEJ repair pathway can be directly connected at the ends of the DNA double-strand breaks, in which the *ku70* and *ku80* proteins play a major role, and the HR repair pathway needs to provide target site for homologous donor DNA fragments (Gorbunova and Levy 1997; Branzei and Foiani 2008). In addition, the possibility of homologous recombination repair will increase by approximately 1000 times if there are nearby homologous DNA fragments during DNA damage repair (Rouet et al. 1994). Based on the above principles, the CRISPR/Cas9 system can use the HR pathway for accurate target editing, such as the introduction of specific point mutations at target sites, insertion of a desired sequence (Feng et al. 2014), or the exact replacement of a sequence with the desired sequence. Recently, Liu et al. (2015) first established a CRISPR/Cas9 system in *T. reesei* by adding homologous arms of different sizes to the left and right of the selectable marker, and demonstrated that when a ≥ 600-bp homology arm is added around the selectable marker, the frequency of homologous recombination is almost 100%, enabling CRISPR/Cas9-mediated gene knockout in *T. reesei*. Subsequently, Zhang et al. (2016) established the MMEJ-CRISPR system in the *ku80* deletion strain *A1160PgpdA-Cas9* (ZC03). The *A1160PgpdA-Cas9* (ZC03) strain eliminates the effects of the NHEJ repair pathway and ensures Cas9 expression. They added short (approximately 35 bp) homology arms near the PAM site to the two sides of the selectable marker HPH and co-transformed with the linear PU6-3-pksP-sgRNA fragment with a mutation efficiency of about 67%. Compared with traditional homologous integration methods with homology arms of at least 500-bp or 1000-bp length (da Silva Ferreira et al. 2006), the data indicates that the CRISPR/Cas9 system with two very short homology arms on the flanks is sufficient to repair the resulting cleavage to achieve the efficient genome editing. Liu et al. (2017) targeted the carbon catabolic repression (CCR) transcription factor *cre-1* in *Myceliophthora thermophila* and *Myceliophthora heterothallica*, demonstrating that, compared with the traditional HR (20%), frequency up to 95% can be achieved by the CRISPR/Cas9 system with repair HR after template co-transformation. The basic method of homologous repair template construction is to add 39–1000 bp upstream and downstream homologous segments of the target gene bracketing the selectable marker, so that the DSBs generated by the CRISPR/Cas9 system can be repaired through the HR pathway and ultimately increase the mutation efficiency of the target gene. Recent studies have shown that circular homologous repair templates are more efficient than linearized templates. This may be due to the fact that circular homologous repair templates cannot directly enter the NHEJ pathway because there are no free ends available for binding to the NHEJ protein and thus may be beneficial for HR repair. In addition, the lack of a free end in a circular gene-targeting substrate also protects the cyclic repair template from exonuclease action, therefore, extending the time that can be used as a repair template (Nodvig et al. 2018).

Interestingly, Shapiro et al. (2018) established a CRISPR-Cas9-based gene-driven array (GDA) platform at *C. albicans*, which inserts two different gRNA in the middle of homologous arms to carry out the genome editing on the adenine biosynthesis gene *ADE2*. When Cas9 cleaves the ORF, the gRNA module is flanked by regions that are homologous to the upstream and downstream sequences of the Cas9-targeting locus, and the entire ORF is deleted and replaced with the targeted gRNA, resulting in a “driven allele.” When the driver-containing haploid cells are mated with wild-type cells, the gRNA-modified locus will initiate another round of cleavage, which converts the incoming wild-type allele into a driver variant. Once the cells contain a functional driver, they can easily convert the heterozygous deletion into a homozygous deletion, facilitating the rapid generation of homozygous deletion mutants in the diploid pathogen. In addition to *C. albicans*, gene-driven platforms may be able to be adapted to other clinically relevant but often overlooked fungal pathogens such as *C. auris* in the future.

## Features of CRISPR/Cas9-induced mutations

Most studies have shown that about half of the CRISPR/Cas9-induced mutations are single-base (mostly A and T) insertions; the rest are small deletions (1–50 bp), and base substitutions of two or more bases insertion are very rare (Feng et al. 2014; Ma et al. 2015). The CRISPR/Cas9 system–mediated single-gene knockouts in the filamentous fungi are mostly single-gene insertions or small fragment deletions (Kujoth et al. 2018). If two or more sites are targeted in a gene or a chromosomal region, a few hundred kilobase fragment deletions may occur between target sites (Zhao et al. 2016).

## Off-target effects

The CRISPR/Cas9 system has the characteristics of high specificity, simplicity in operation, and high efficiency and has been widely used in the biological research and biomedical application. However, the off-target effects of the CRISPR/Cas9 system are a major concern. Previous studies have shown that there are up to five mismatch target genes for each gRNA (Cradick et al. 2013; Fu et al. 2013). Off-target effects are mainly divided into the following types: (1) in most cases, Cas9/gRNA does not recognize more than three mismatched DNA sites; (2) Cas9/gRNA does not recognize and edit DNA sites with any number of mismatches (within 10–12 bp) near the PAM; (3) the higher the Cas9/gRNA concentration, the greater the possibility of off-target effects (Hsu et al. 2013; Mali et al. 2013b; Pattanayak et al. 2013). In order to avoid off-target effects, Wang and colleagues designed plasmid DNA, which may remove the inserted Cas9 gene after genome editing (Wang et al. 2016). Recently, Nagy et al. (2017) developed a CRISPR-Cas9 system specifically for transient expression. This system assembles Cas9 protein and sgRNA to form a stable RNP in vitro, and transfers the complex to fungal protoplasts by PEG- or electroporation-mediated methods for genome editing. This method enables the in vitro evaluation of target cleavage efficiency of different sgRNAs, so efficient sgRNAs can be selected for research. Furthermore, the transformation of RNP mitigates the possibility of integration of genetic material into non-targeted regions of the genome. Methods based on next-generation sequencing technologies such as GUIDE-seq (Tsai et al. 2015), Digenome-seq (Kim et al. 2015), and ChIP-seq (Kuscu et al. 2014) can be also used to identify the off-target site. The above strategies can be used to provide guidance for increasing the specificity of the CRISPR/Cas9 system in filamentous fungi.

## Application of CRISPR/Cas9 in industrial strains

Enzymes produced by filamentous fungi play an important role in industrial production, for example, in paper, food, feed, textiles, and detergents. Filamentous fungi are used in food fermentation and industrial production of recombinant proteins, and they also serve as hosts for industrially valuable secondary metabolites. Obviously, the production of various important enzymes by industrial strains has become the focus of industry and academia. However, wild-type strains cannot produce the desired enzymes at an industrial level. Therefore, genetic engineering is needed to enhance these fungi involved in industrial production but traditional continuous modification of multiple genes using a limited number of selection markers is laborious. To solve this problem, researchers have developed different methods to increase their enzyme yield but most methods are only suitable for a limited number of fungi. With the development of more and more fungal genome sequences and omics data, the study of industrial fungi has brought a wider range of genetic manipulation opportunities, and the use of CRISPR/Cas9 genome editing techniques in fungi producing industrial enzymes greatly improves the ability of these fungi to produce industrial enzymes.

For example, *T. reesei* is the main source of the most widely used commercial lignocellulolytic enzyme preparations, a potential cell factory for heterogeneous protein and secreting proteins, often used to produce a variety of enzymes or metabolites. Initially, in order to establish the CRISPR/Cas9 genome editing technology in *T. reesei*, Liu et al. (2015) optimized the Cas9 gene of *S. pyogenes* and linked it to the nuclear localization signal SV40. The *lae1* and *vib1* genes were successfully knocked out by in vitro synthesis of gRNA. Later, Leynaud-Kieffer et al. (2019) used the CRISPR/Cas9 genome editing technology to knock out the *SxlR* gene, a negative regulator of xylanase activity. The results indicate that deletion of the *SxlR* gene results in a significant increase in gene expression encoding the *GH11* endoxylanase, whereas overexpression of this gene results in a decrease in xylanase activity but does not affect cellulase activity. Industrial fungi producing cellulase also include *A. oryzae*. In Japan, *A. oryzae* is an important industrial filamentous fungus for traditional fermentation, production of enzymes and heterologous proteins. In order to efficiently produce these enzymes and compounds, it is necessary to genetically engineer the *A. oryzae* strain but continuous (single) changes to multiple genes using conventional methods are time consuming. In 2016, Katayama et al. (2016) developed the CRISPR/Cas9 method for mutagenesis of *A. oryzae* but the efficiency of this method is very low. In order to improve the editing efficiency of the CRISPR/Cas9 genome editing technology in *A. oryzae* strains, Katayama et al. (2019) used a plasmid containing the AMA1 autonomously replicating sequence to increase the mutation efficiency of *A. oryzae* wild and industrial strains to 50–100%. The AMA1 plasmid was present in multiple copies in the *A. oryzae* strain, thereby increasing the expression of Cas9 and sgRNA, and ultimately increasing the mutation efficiency of the *A. oryzae* strain. In addition, AMA1-based plasmids are discarded when there is no resistance to stress so this approach avoids the impact of the CRISPR/Cas9 system on the strain. In theory, the AMA1-based CRISPR/Cas9 genome editing technology will allow for infinite rounds of genetic engineering. The AMA1 autonomously replicating sequence can be expressed in a variety of filamentous fungi, including not only *Aspergillus* species but also *P. chrysogenum*, *Talaromyces atroroseus*, and *Gaemannomyces graminis*.

In addition to its use in these enzyme-producing fungi, the CRISPR/Cas9 genome editing technology can also be used in other industrial fungi. As we all know, *Ashbya gossypii* is an industrial fungus used in the production of vitamin B_2_. To date, a large number of genetic engineering and biotechnology tools are available for *A. gossypii*. For example, Cre-loxP technology is applied to *A. gossypii*. However, a large number of modifications greatly reduced the ability of the strain to produce spores and a large number of loxP scars were produced after multiple rounds of operation (Ledesma-Amaro et al. 2015). In 2019, Jiménez et al. (2019) knocked out the *ADE2* gene of *A. gossypii* using a one-vector CRISPR/Cas9 system controlled by the yeast promoter *SNR52*, and verified the presence of an 8-bp deletion in the *ade2* mutant by DNA sequencing. The stability of this mutation was then confirmed because no phenotypic changes were observed after five-spore formation. Thereafter, in order to verify the stability of the system, Jiménez et al. (2019) transferred the CRISPR/Cas9 vector containing the sgA754-dA754 fragment into strain A754. PCR identification and DNA sequencing confirmed that the system can eliminate loxP scars in *A. gossypii*. These examples above show that the CRISPR/Cas9 system has been effectively applied to industrial fungi, and the use of the CRISPR/Cas9 genome editing system can further enhance the applicability of these industrial fungi.

## Conclusion and perspectives

Compared with ZFN and TALEN systems, the CRISPR/Cas9 system has several advantages. (1) The CRISPR/Cas9 system is widely used: the requirement for the selection of target sites in this system is to have a PAM sequence, which is widely present in the genome and can find several target sites in any gene, so that almost all genes can be edited. (2) The CRISPR/Cas9 system consists of simple components: sgRNA and Cas9 proteins are the two major components of the system. They can be placed on a transformation vector as needed, or placed on different transformation vectors, or assembled directly in vitro. (3) The CRISPR system can reduce the selection of markup applications: For example, a limiting factor in mutagenesis in *A. fumigatus* stems from a limited number of dominant selection markers. When we need to mutate three or more genes in a row, the only selection markers available for *A.* fumigatus are *hph*, *ble*, and *ptrA*. The CRISPR/Cas9 system can edit multiple genes at the same time, and it is possible to obtain mutants with multiple site mutations in one transformation, which greatly improves the efficiency of genome editing.

However, at present, there are still deficiencies in this technology, such as off-target effect, needs to improve editing efficiency, and need to construct a carrier system that is applicable to various fungi. There is also a risk that the system will not stop the genome editing function after the error gene is repaired, but may continue to modify the normal gene to cause off-target effects. To address this problem, Nagy et al. (2017) developed the CRISPR-Cas9 system specifically for transient expression. This system not only evaluates the target cleavage efficiency of different sgRNAs in vitro but also reduces the possibility of integration of genetic material into non-targeted regions of the genome. These advantages greatly reduce off-target effects of the CRISPR system.

Cho et al. (2014) has proposed that Cas9 inhibitory protein can be used to disable the runaway editing function by inhibiting its activity when Cas9 completes genome editing, but it is in its early stage and further experiments are needed to verify and identify better techniques. Based on the principle of DNA double-strand break and chromosomal translocation, Yin et al. (2019) developed a new method PEM-seq with higher sensitivity and comprehensive and quantitative evaluation of gene editing based on high-throughput sequencing methods. Compared with previous methods based on second-generation sequencing to assess the off-target activity of Cas9, PEM-seq not only can sensitively identify the off-target sites of Cas9 but also can accurately quantify the cutting efficiency of CRISPR/Cas9 at the target site, thus finding more efficient Cas9 cutting site. Although these methods have not yet been established in fungi, with the continuous development and improvement of CRISPR/Cas9 technology, it is certain that the technology will play a greater role in the future research for genetic modification of filamentous fungi.
